# Prevalence of human herpesviruses in biliary fluid and their association with biliary complications after liver transplantation

**DOI:** 10.1186/s12876-019-1033-x

**Published:** 2019-06-27

**Authors:** Conrad Rauber, Katja Bartelheimer, Taotao Zhou, Christian Rupp, Paul Schnitzler, Peter Schemmer, Peter Sauer, Karl Heinz Weiss, Daniel Nils Gotthardt

**Affiliations:** 1 Department of Gastroenterology and Hepatology, University Hospital Heidelberg, Heidelberg, Germany; 2 Department of General, Visceral and Transplant Surgery, University Hospital Heidelberg, Heidelberg, Germany; 3 Department of Virology, University Hospital Heidelberg, Heidelberg, Germany; 4 Department of Surgery, Division of Transplant Surgery, Medical University of Graz, Graz, Austria; 5 INSERM U1015, Gustave Roussy Comprehensive Cancer Institute, Villejuif, France

**Keywords:** Herpesvirus, HHV-6, Bile, Stricture, Non-anastomotic stricture, Liver transplantation, Complication

## Abstract

**Background:**

Beta-herpesviruses are common opportunistic pathogens that cause morbidity after liver transplantation (LT).

**Methods:**

Objective of the study was to evaluate the prevalence and correlation of herpesviruses in bile, blood and liver tissue and to investigate their association with biliary complications and retransplantation (re-LT) free survival after LT.

The study design is a single-center case-control study. We performed quantative polymerase chain reaction (qPCR) for herpesvirus 1–8 DNA in bile, blood and liver tissue of 73 patients after first LT and analyzed their clinical courses retrospectively.

**Results:**

The median follow-up was 48 months (range 2–102), during which a total of 16 patients underwent re-LT and 11 patients died. Of the patients, 46.5% received valganciclovir prophylaxis at the time of bile sample acquisition. Cytomegalovirus (CMV) (18.3%), human herpesvirus 6 (HHV-6) (34.2%), human herpesvirus 7 (HHV-7) (20.5%) and Epstein-Barr virus (EBV) (16.4%) were highly prevalent in bile after LT, while herpes simpex virus 1 and 2 (HSV-1, HSV-2), varicella-zoster virus (VZV) and human herpesvirus 8 (HHV-8) were not or rarely detected in bile. Valganciclovir prophylaxis did not reduce the prevalence of HHV-6 and HHV-7 in bile, but it did reduce the presence of CMV and EBV. The presence of HHV-6 in bile was associated with non-anastomotic biliary strictures (NAS) and acute cellular rejection (ACR).

**Conclusions:**

CMV, EBV, HHV-6 and HHV-7 are more prevalent in biliary fluid than in liver biopsy or blood serum after LT. HHV-6 and HHV-7 might be associated with biliary complications after LT. Biliary fluids might be an attractive target for routine herpesvirus detection.

## Background

Liver transplantation (LT) is to date the only curative option for patients with end-stage liver disease. Liver transplantation requires lifelong immunosuppression to prevent allograft rejection and subsequent graft failure. Immunosuppression predisposes solid organ recipients to various opportunistic infections. The role of many of these opportunistic pathogens in the development of complications after LT is unknown. Human herpesviruses (HHV) 1–8 are enveloped, double-stranded DNA and human host specific viruses that proliferate in lymphocytes and neuronal or epidermal cells. They can persist lifelong in the host and reactivate under circumstances of immunosuppression. Active disease can be detected by polymerase chain reaction (PCR) of HHV 1–8 DNA [1]. The incidence of Cytomegalovirus (CMV), HHV-6 and HHV-7 in blood after LT has been reported as high as 70, 33 and 42%, respectively [2]. CMV is the most common infectious pathogen after LT [3] and can infect various organs, including the allograft itself. CMV in bile has been associated with early graft loss and biliary complications after LT [4]. HHV-6 and HHV-7, together with CMV (HHV-5), comprise the group of beta-herpesviruses. Human herpesvirus 6 has been linked to hepatitis post LT, but most HHV-6 infections after LT are asymptomatic [5, 6]. The presence of HHV-6 and HHV-7 viremia in blood after LT has inconsistently been linked to reduced graft survival. However, detecting HHV-6 DNA in liver biopsies has been associated with graft hepatitis and reduced graft survival [7–9]. Epstein-Barr virus (EBV) and CMV detection in liver biopsies had no effect on overall survival after LT [7]. To prevent the reactivation of CMV after LT, patients receive valganciclovir chemoprophylaxis for three to 6 months after LT [10]. For HHV-6 and -7, there are no recommendations for prevention. In vitro, HHV-6 and HHV-7 are less susceptible to ganciclovir than CMV [11]. The objective of this study was to examine the association between herpesviruses in different body fluids and tissues after LT and to elucidate their role in graft complications after LT.

## Methods

### Study population

**Fig. 1:**
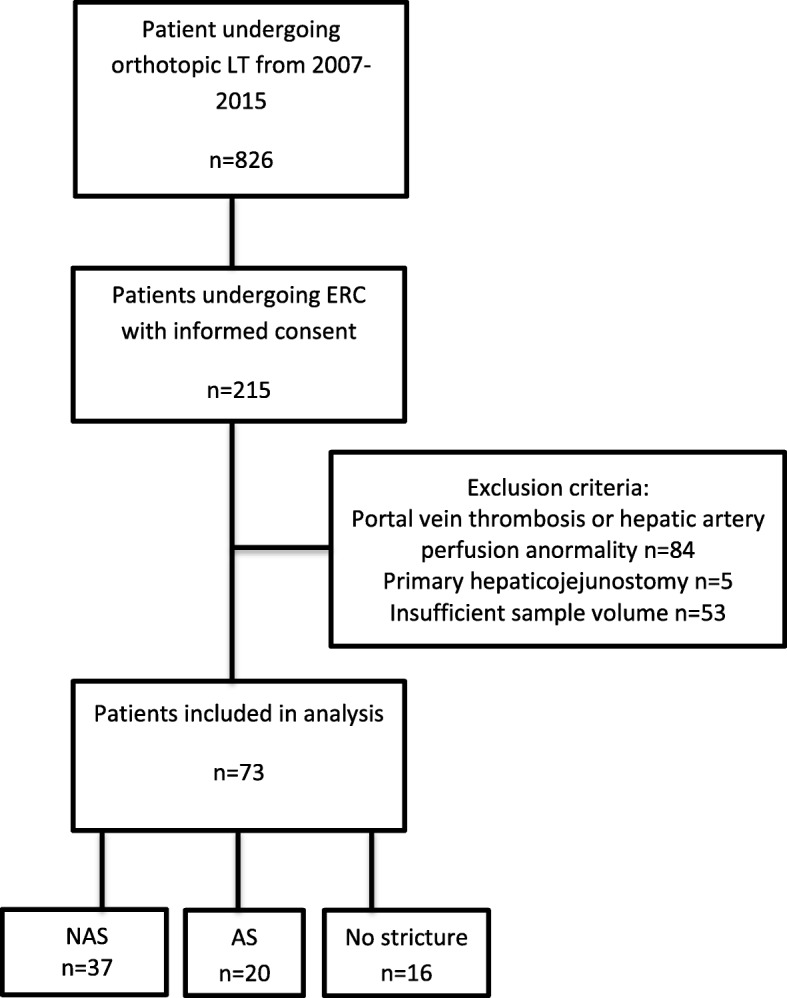
Flowchart of patients’ inclusion in the analysis. AS- anastomotic stricture, LT- liver transplantation, NAS – non-anastomotic stricture

In this single-center case-control study, patients were selected retrospectively out of all adult patients who underwent a first liver transplantation at the University Hospital Heidelberg between January 2007 and July 2015, for which period bile samples were available in our biobank (*n* = 215). Exclusion criteria were death within 30 days after transplantation, risk factors for ischemic bile duct injury such as hepatic artery stenosis, portal vein stenosis or portal vein thrombosis (*n* = 84), insufficient sample volume (*n* = 53) and primary hepaticojejunostomy (*n* = 5). Of the remaining 73 patients, we included bile samples for 37 patients with NAS, 20 patients with AS and 16 without stricture (Fig. 1). 19 patients had developed acute cellular rejection after LT of which 13 were diagnosed before and 6 after ERC.

### Sample acquisition

Bile samples were retrieved from ERC examinations, immediately frozen and stored at a temperature of − 80 °C. Indications for ERC in all patients were abnormal liver laboratory values. Indications for liver biopsies were suspected allograft rejection or infection. Samples were formalin-fixed, paraffin-embedded and stored appropriately. Blood samples were taken routinely for check-up or if clinically indicated and stored at − 20 °C.

### Immunosuppressive and chemoprophylactic regime

All patients received corticosteroids for 6 months after transplantation. Additionally, a calcineurin inhibitor (CNI) (ciclosporin or tacrolimus) was administered, beginning within the first days after transplantation. Mycophenolate (MMF) was either started directly after transplantation (de novo), or after 21 days (delayed). Infectious complications led to discontinuation of MMF, according to internal clinical guidelines. Prophylaxis for CMV infection with valganciclovir was administered to all patients within the first 3 to 6 months after transplantation at a dose of 900 mg once daily (both CMV IgG seropositive donor/recipient matches did not receive CMV prophylaxis).

### Transplant allocation criteria

All patients were transplanted within the Eurotransplant allocation system in Germany [12]. Patients were transplanted according to the model of end-stage liver disease (MELD) score, which is based on the recipient’s kidney function, coagulation time and serum bilirubin and ranges from 6 to 40 [13]. Patients transplanted for hepatocellular carcinoma were granted an exceptional MELD score (eMELD) in which the allocation is based on waiting time [14].

### Clinical follow-up

The clinical records of the patients were reviewed in our electronic patient database (i.s.h. med, SAP, Germany). We recorded demographic data, underlying diseases and the reasons for transplantation, immunosuppressive regimen and antiviral prophylaxis, infectious complications such as herpesvirus infections, biliary complications, incidence of acute or chronic graft rejection, information on graft, donor and recipient hepatitis- and CMV-status and recipient post- and perioperative values. All patients were followed until death or retransplantation or until October 1, 2015.

### Detection of herpesvirus-DNA in bile, blood and liver tissue

DNA extraction. Herpesvirus-DNA was extracted from 400 μl of bile and 200 μl of serum using QIAamp DNA blood mini-kit (Qiagen, Germany). To extract DNA from the formalin-fixed paraffin-embedded liver biopsies, the QIAamp DNA FFPE tissue kit (Qiagen, Germany) was used. Both serum and bile samples were stored at − 80 °C, and extracted DNA was temporarily stored at − 20 °C. The DNA concentration after extraction was measured with a Colibri microvolume spectrometer (Titertek-Berthold, Germany).

Quantitative real time polymerase chain reaction (qPCR). Virus-specific r-gene quantification kits (Biomerieux, France) were used to prepare the extracted DNA for quantification. The kit contains an internal control that was added to the samples before DNA extraction to monitor the extraction process and the presence of amplification inhibitors. Appropriate negative controls were performed to check for contamination along the extraction and amplification process. Quantification standards were available for HHV 1–6 allowing the measurement of the viral DNA load. The detection of HHV-7 was qualitative, and the HHV-7 viral count could not be quantified. PCR was performed using the LightCycler 480 (Roche Diagnostics, Switzerland).

### Statistical analysis

Descriptive statistics were used to characterize patients’ demographics and clinical variables. Continuous variables are expressed as mean values with standard deviation (SD), non-normally distributed variables as median values with range and categorical variables as number and percent. A chi-square test was used to compare the frequency of differences in patient characteristics, complications and medical regimens according to herpesvirus detection in bile. Statistical data was analyzed using SPSS (SPSS 22.0 Inc., USA).

## Results

### Total population

The clinical baseline characteristics of LT subjects are summarized in Table 1.

**Table 1:** Characteristics of patients who underwent LT. Data is given as mean (± SD), median (range) or number (%), as appropriate

Number of patients	73
Gender recipient, female	15 (20.5%)
Gender donor, female	36 (49.3%)
Recipient age, years, (median, range)	56 (30–69)
Donor age, years, (median, range)	67 (21–88)
Follow-up, months, (median, range)	48 (2–102)
Child-Turcotte-Pugh score (A/B/C) at LT, n	25/16/31
Valganciclovir prophylaxis at time of ERC	34 (47.1%)
Time of ERC, months since LT, (median, range)	3.4 (0.3–73)
Lab/eMELD at LT (mean ± SD)	27.0 ± 8.7
Cold ischemia time, hours (mean ± SD)	10.0 ± 2,64
Indication	
Alcoholic cirrhosis	23 (31.5%)
Hepatitis B	2 (2.7%)
Hepatitis C	11 (15.1%)
HCC	15 (20.5%)
PSC	2 (2.7%)
Cryptogenic	7 (9.6%)
Other	13 (17.8%)
Death during follow-up	11 (15.1%)
Retransplantation	16 (21.9%)
Ciclosporin de novo	51 (69.9%)
Tacrolimus de novo	20 (27.4%)
Mycophenolate mofetil	61 (83.6%)
Anastomotic stricture	34 (46.6%)
Non-anastomotic stricture	37 (50.7%)
Acute rejection	19 (26.0%)

de novo: administered directly after LT; *eMELD* exceptional model of end-stage liver disease, *ERC* endoscopic retrograde cholangiopancreatography, *HHC* hepatocellular carcinoma, *LT* liver transplantation, *labMELD* laboratory model of end-stage liver disease, *PSC* primary sclerosing cholangitis

The median follow-up was 48 months (range 2–102) (Table 1). A total of 16 patients underwent re-LT at a median time of 11 months (range 1–42), and 11 patients died during follow-up after a median time of 15.2 months (range 7–37), with follow-up terminating at the combined endpoint of re-LT or death. The average age of LT recipients was 56 (range 30–69) years. Recipients were predominantly male (79.5%), and the most frequent indication for LT was alcoholic cirrhosis (34.5%). Donors were slightly older with a mean age of 67 (range 21–88) years, while gender was evenly distributed (48.6% female). The ERC from which bile was retrieved occurred at a median of 3.4 months (range 0.3–73) after LT. At time of ERC, 46.5% of patients were receiving valganciclovir as cytomegalovirus prophylaxis (900 mg per day). All patients received immunosuppression at time of ERC, and all but two patients received calcineurin inhibitors (CNI) de novo (69.9% ciclosporin, 30.1% tacrolimus), while 83.6% received additional mycophenolate mofetil. Neither the time between LT and ERC nor the immunosuppressive regimen at ERC significantly influenced the rate of herpesvirus positivity in bile.

We tested ERC bile samples for herpesvirus 1–8. For 42 patients concordant serum samples were available (median time of 8 days before or after ERC, *n* = 42). For beta-herpesviruses (CMV, HHV-6, HHV-7), we also tested available liver biopsies (median time of 38 days before or after ERC, *n* = 53). Rates of herpesvirus positivity are shown in Table 2. HSV-1 and HSV-2 were rarely detected in bile (4.1 and 0%, respectively), while there was a significant rate of detection in serum for at least HSV-1 (14.3%). Varicella-zoster virus appeared to be rare after LT in serum (0%) and bile (2.4%), with only a single positive bile sample. EBV was often detected in bile (16.4%) and serum samples (9.5%). Cytomegalovirus is a pathogen known to be relevant after LT. It was significantly more prevalent in bile (18.3%) than in serum samples (2.4%) or liver tissue (0%); this finding has been described before [4]. HHV-6 was highly prevalent in bile (34.2%) and liver biopsies (15.1%) but was rarely found in serum (2.4%). The DNA copy number in bile was rather low, with a median copy number of 12.6 copies/ml in HHV-6 positive patients whereas four patients had biliary HHV-6 DNA titers > 1000 copies/ml. The median HHV-6 copy number in liver biopsy was 20 copies/ml among those patients with amplifiable HHV-6 DNA. Only one patient in the liver biopsy group had a high copy number, that was over 1000 copies/ml. Liver biopsy and bile HHV-6 DNA copy number did not correlate significantly (Pearson correlation *r* = − 0.026, *p* = 0.87). HHV-7 was also highly prevalent in bile samples (20.5%) but was not detected in serum (0%) and rarely in liver biopsy (3.8%) samples. HHV-6 and HHV-7 in bile correlated significantly (Pearson correlation *r* = 0.28, *p* = 0.02). HHV-6 reactivation due to HHV-7 infection has been described previously [15].

**Table 2:** Herpesvirus prevalence after liver transplantation

	Bile	Serum	Liver Biopsy
HSV-1	3/73 (4.1%)	6/42 (14.3%)	
HSV-2	0/73 (0%)	1/42 (2.4%)	
VZV	1/72 (1.4%)	0/42 (0%)	
EBV	12/73 (16.4%)	4/42 (9.5%)	
CMV	13/71 (18.3%)	1/42 (2.4%)	0/51 (0%)
HHV-6	25/73 (34.2%)	1/42 (2.4%)	8/53 (15.1%)
HHV-7	15/73 (20.5%)	0/42 (0%)	2/53 (3.8%)
HHV-8	1/64 (1.6%)		

*CMV* cytomegalovirus, *EBV* Epstein-Barr virus, *HHV* human herpesvirus, *HSV* herpes simplex virus, *VZV* varicella-zoster virus

Of the 53 patients where HHV-6 was tested in both bile and biopsy, 29/53 (54.7%) tested concordantly negative, while 4/53 (7.5%) tested concordantly positive, 16/53 (30.2%) tested positive in bile but not in biopsy and 4/53 tested positive in biopsy but not in bile (7.5%) (Table 3). In chi-square test bile and biopsy positivity for HHV-6 were not significantly associated (*p* = 0.35), but those that tested concordantly positive where taken on significantly closer timepoints (median time between bile sample and biopsy was 3 days in concordantly positive bile and biopsy samples vs. median time of 21 days in non-concordant bile and biopsy samples).

**Table 3:** Concordance of HHV-6 positivity in bile and liver biopsy

HHV-6 detection		Bile		
		neg.	pos.	Total
Biopsy	neg.	29	16	45
	pos.	4	4	8
	Total	33	20	53

Chi-square = 0.35

We correlated HHV-6 with baseline characteristics and compared the prevalence of HHV-6 positivity according to complications after liver transplantation. The labMELD score at the time of transplantation was significantly higher patients positive for HHV-6 in bile (16.4 vs. 22.5, *p* = 0.02, Table 4), while the eMELD score (36 patients) was similar in HHV-6 in bile positive and negative patients (27.6 vs. 27.3, Table 4).

**Table 4:** Characteristics of patients according to HHV-6 positivity in bile. Comparison of patients based on the main primary outcome parameter of retransplantation-free survival. Data is given as mean (± SD), median (range) or number (%), as appropriate

	HHV-6 negative, *n* = 48 (65.7%)	HHV-6 positive, *n* = 25 (34.2%)	p
Gender, female (%)	8/48 (16.7%)	7/25 (28.0%)	0.20
Recipient Age (median, range), years	55 (30–69)	59 (41–66)	0.36
Follow up (median, range), months	39.5 (2.0–91.2)	20.7 (0.6–102.9)	0.78
Time of ERC (median, range), months	3.5 (0.3–73.6)	2.4 (0.3–22.1)	0.38
Donor age (median, range), years	60 (21–88)	61 (27–82)	0.65
eMELD at LT, points (mean ± SD)	27.6 (± 5.3)	27.3 (± 4.9)	0.87
labMELD at LT, points (mean ± SD)	16.4 (± 9.5)	22.5 (± 12.2)	0.02
Cold ischemia time,hours, (mean ± SD)	9.8 (± 2.6)	10.4 (± 2.7)	0.37
AS	25/48 (48.1%)	9/25 (36.0%)	0.17
NAS	20/48 (41.7%)	17/25 (68.0%)	0.03
Acute rejection	8/48 (16.6%)	11/25 (44.0%)	0.01
CMV positivity in bile	10/48 (20.8%)	3/23 (13.0%)	0.33
HHV-7 positivity in bile	6/48 (12.5%)	9/25 (36.0%)	0.02
HHV-6 positivity in liver biopsy	4/33 (12.1%)	4/20 (20.0%)	0.30
HHV-6 positivity in serum	0/30 (0%)	1/11 (9.1%)	0.29
AP (U/ml, SD)	194.1 (± 125.4)	195.6 (± 153.8)	0.96
Bilirubin (mg/ml, SD)	6.2 (± 9.3)	7.6 (± 8.8)	0.52
AST (U/ml, SD)	100.9 (± 102.9)	144.9 (± 192.5)	0.21
Leukocytes (cells/nl, SD)	6.2 (± 2.7)	7.7 (± 5.5)	0.12

AP: alkaline phosphatase, AS: anastomotic biliary stricture, AST: aspartate aminotransferase, CMV: cytomegalovirus, eMELD: exceptional model of end-stage liver disease, ERC: endoscopic retrograde cholangiopancreatography, HHV: human herpesvirus, LT: liver transplantation,labMELD: laboratory model of end-stage liver disease, NAS: non-anastomotic biliary stricture, SD: standard deviation

HHV-6 positivity in bile was also associated with the occurrence of allograft complications after LT. Non-anastomotic strictures (NAS) of the bile duct (41.7% vs. 68.0%, *p* = 0.03, Table 4) and acute cellular rejection of the liver graft (16.6% vs. 44.0%, *p* = 0*.*01, Table 4) were significantly more common in patients with HHV-6 positivity in bile. Patients positive for HHV-6 in bile were significantly more likely to also be positive for HHV-7 in bile (12.5% HHV-7 pos. in HHV-6 neg. vs. 36.0% HHV-7 pos. in HHV-6 neg., *p* = 0.02, Table 4). Surprisingly, HHV-6 positivity in bile did not correlate with HHV-6 positivity in serum samples or liver biopsy (Table 4). In addition, for the other herpesviruses, no correlation was observed between biliary positivity and biopsy or serum positivity (data not shown).

We assessed whether our patients had received valganciclovir as CMV prophylaxis at time of ERC. Valganciclovir was routinely administered for 6 months after LT to prevent CMV reactivation in the liver allograft. Valganciclovir prophylaxis did reduce the incidence of CMV positivity in bile (9.1% vs. 27.8%, *p* = 0.04, Table 5), but valganciclovir prophylaxis had no effect on the rate of HHV-6 (38.2% vs. 27%, *p* = 0.25, Table 5) or HHV-7-positivity in bile (17.6% vs. 18.9%, *p* = 0.9, Table 5). The rate of EBV-positivity in bile (8.9% vs. 24.3%, *p* = 0.07, Table 5) was numerically reduced but did not reach statistical significance.

**Table 5:** Valganciclovir and herpesvirus reactivation. Data is given as number (%)

	LT recipients (*n* = 71)		
	Valganciclovir prophylaxis (*n* = 34)	No Valganciclovir prophylaxis (*n* = 37)	p
EBV positive	3/34 (8.9%)	9/37 (24.3%)	0.07
CMV positive	3/33 (9.1%)	10/36 (27.8%)	0.04
HHV-6 positive	13/34 (38.2%)	10/37 (27%)	0.25
HHV-7 positive	6/34 (17.6%)	7/37 (18.9%)	0.9

*CMV* cytomegalovirus, *EBV* Epstein-Barr virus, *HHV* human herpesvirus, *HSV* herpes simplex virus, *LT* liver transplantation, *VZV* varicella-zoster virus

**Fig. 2:**
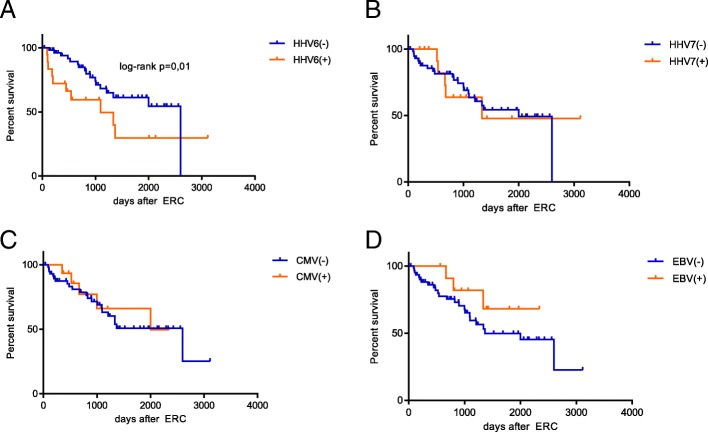
Retransplantation free survival after ERC according to herpesvirus positivity in bile. Comparison of retransplantation free survival after ERC according to herpesvirus positivity in bile, comparison with log-rank. A Human herpesvirus 6 (HHV-6) B Human Herpesvirus 7 (HHV-7) C Cytomegalovirus (CMV) D Epstein-Barr-Virus (EBV)

Patients that tested HHV-6 positive in bile were numerically more likely to die or undergo re-LT after ERC. Median survival after ERC for HHV-6 in bile positive versus HHV-6 in bile negative patients was 36.7 vs 86.7 months respectively (log-rank *p* = 0.01, Fig. 2). There was no statistically significant difference noted for any other herpesvirus (Fig. 2). In univariate cox proportional hazard model HHV-6 in bile was negatively associated with retransplantation free survival (hazard ratio 2.72, p = 0.01). We included all know risk factors in the analysis and included them in a step up approach, when *p* < 0.1. This led to the inclusion of time from LT to ERC, NAS, valganciclovir at ERC and HHV-7 in bile. In multivariate analysis HHV-6 in bile lost its significant negative association with retransplantation free survival (hazard ratio 2.15, p = 0.07), but remained the strongest risk factor for retransplantation or death among the included (Table 6).

**Table 6:** Uni- and multivariate analysis of risk factors for graft loss or death

	Univariate		Multivariate	
	Hazard ratio (95% CI)	p	Hazard ratio (95% CI)	p
HSV-1 positivity (bile)	2.34 (0.70–7,80)	0.17		
HSV-1 positivity (serum)	0.43 (0.06–3.49)	0.39		
EBV positivity (bile)	0.82 (0.28–2.38)	0.72		
EBV positivity (serum)	2.18 (0.47–10.02)	0.32		
CMV positivity (bile)	0.21 (0.29–1.58)	0.13		
HHV-6 positivity (bile)	2.72 (1.25–5.90)	0.01	2.15 (0.94–4.95)	0.07
HHV-6 positivity (liver biopsy)	1.02 (0.30–3.46)	0.98		
HHV-7 positivity (bile)	2.57 (1.14–5.78)	0.02	2.03 (0.81–5.12)	0.13
Valganciclovir at time of ERC	1.78 (0.97–3.26)	0.07	0.99 (0.48–2.07)	0.87
Time between LT and ERC [months]	0.93 (0.86–1.01)	0.08	0.94 (0.79–1.12)	0.27
NAS vs. AS/control	1.74 (0.99–3.05)	0.05	0.94 (0.79–1.12)	0.47
Recipient age, years	1.02 (0.97–1.07)	0.49		
Donor age, years	1.02 (0.99–1.04)	0.18		
MELD score at LT	0.94 (0.81–1.01)	0.12		
Cold ischemia time	0.96 (0.83–1.11)	0.39		

*AS*, anastomotic biliary stricture, *CMV* cytomegalovirus, *EBV* Epstein-Barr virus, *HHV* human herpesvirus, *ERC* endoscopic retrograde cholangiopancreatography, *HSV* herpes simplex virus, *LT* liver transplantation, *MELD* model of end-stage liver disease, *NAS* non-anastomotic biliary stricture, *VZV* varicella-zoster virus

## Discussion

Biliary fluids can routinely be assessed after ERC but are rarely subject to scientific investigation. This is the first single-center case-control study to investigate herpesvirus 1–8 prevalence in human bile samples and its association with biliary complications after LT. We found a high prevalence of CMV, HHV-6 and HHV-7 in biliary fluids in LT patients both with and without biliary complications. The rate of positivity of HHV-6 correlated with poor re-LT-free survival after ERC. The persistence of beta-herpesviruses in epithelia after LT has been described frequently [16]. Cytomegalovirus in bile has already been implicated in biliary lesion formation after LT, but its significance remains controversial [4, 17, 18]. Interestingly, in our study, the rates of detection of CMV, HHV-6 and HHV-7 in bile have been considerably higher than in serum or liver biopsies (Table 2). In bile we found the highest prevalence for HHV-6 (34.2%) and HHV-7 (20.5%) followed by CMV (18.3%) and EBV (16.4%) (Table 2). Presence of HHV-6 and HHV-7 often coincided possibly due to viral coactivation, as 9/15 patients positive for HHV-7 in bile also tested positive for HHV-6 [19]. We found that patients with NAS and ACR were much more likely to test positive for HHV-6 and HHV-7 in bile than patients of the control group (Table 4). Interestingly, neither HHV-6 nor HHV-7 in liver biopsy or serum were associated with biliary complications after LT. In Kaplan-Meier analysis HHV-6 was significantly associated with death and retransplantation after ERC. This significance was lost however in multivariate cox proportional hazard analysis after controlling for known risk factors such as type of stricture in the multivariate analysis (Fig. 2 and Table 6), however HHV-6 in bile remained the strongest risk factor for decreased re-LT free survival among the included. If HHV-6 in bile is causally related to biliary strictures and thus graft survival or a mere bystander of damage of the biliary epithelium remains to be determined. We are not the first group to report the relation of HHV-6 and decreased graft survival. In a cohort of pediatric LT recipients chromosomally integrated HHV-6 DNA was associated with worse graft survival [20]. HHV-6 has also been associated with worse LT graft survival in an adult population after diagnosis of graft hepatitis with higher intrahepatic HHV-6 amplification levels [7]. The mechanism for this association remains unclear. However HHV-6 has been described to exert immunomodulatory effects such as increased secretion of interferon gamma, tumor-necrosis factor alpha or CCL5 [21–24]. HHV-6 has also been implicated in numerous autoimmune diseases such as multiple sclerosis, connective tissue disorders or autoimmune thyroiditis in part through molecular mimicry [25–29]. HHV-6 could thus exert immunomodulatory effects in the liver allograft leading to a host versus graft reaction leading to further damage of the biliary ducts.

The preexisting biliary damage of the donor organ at time of transplantation has been shown to predict subsequent risk for biliary complications [30]. We therefore assessed labMELD and cold ischemia time as established risk factors for mortality and associated with early allograft loss, which were evenly distributed in our study.

Increased recipient morbidity at time of transplantation likewise seems to play a role for biliary HHV-6 infections. We recorded labMELD score at transplantation as a surrogate marker for recipient morbidity at transplantation [31]. Patients positive for HHV-6 in bile had a higher median labMELD (16.4 vs. 22.5, *p* = 0.02, Table 4) but not a higher eMELD score at LT. Patients with a higher labMELD score at the time of transplantation presented more severe hepatic and renal impairment. This precondition might predispose patients to opportunistic infections and, in our study, might in part explain the increased prevalence of herpesvirus infections. The association of HHV-6 with graft complications and reduced survival poses the important question of possible prevention of HHV-6 reactivation/infection. Interestingly, valganciclovir prophylaxis did not reduce the incidence of HHV-6 prevalence nor the mean viral count in bile (38.2% vs. 27%, *p* = 0.25, Table 5). This might be due to a lower responsiveness of HHV-6 and HHV-7 than CMV to valganciclovir or ganciclovir treatment [32]. In a study by Humar et al. of CMV, HHV-6 and HHV-7 coinfected patients, valganciclovir treatment did reduce the CMV but not the HHV-6 and HHV-7 viral load [32]. Consistent in our study CMV prophylaxis was sufficient in our patients to prevent CMV infection. This might explain why in our study CMV in bile was not related to NAS, contrasting with prior data published by our group [4]. In the current study, bile retrieval was undertaken early after LT while many patients (47.8%) were under CMV prophylaxis. When excluding patients on prophylactic therapy, there was a numerical though statistically insignificant higher prevalence of non-anastomotic strictures in patients who tested positive for CMV in bile (50% vs. 33%, ns).

Two important findings conclude our study. The first is the striking discrepancy of herpesvirus detection of the different compartments (bile, serum, tissue). Currently, blood serum is used for routine HHV-6 detection; indeed biliary samples might be more sensitive and specific for HHV-6 detection in the liver allograft. This finding is not limited to herpesviruses but might extend to all DNA-viruses and bacteria. We thus hypothesize that testing biliary fluid might mirror local infective processes of the hepatobiliary compartment with a higher sensitivity than serum or biopsy specimen. The second important finding is the correlation between locoregional HHV-6 (and possibly CMV in the later course) detection and severe liver allograft complications. We further hypothesize that routine CMV prophylaxis with valganciclovir is not sufficient to prevent HHV-6 (and HHV-7) infection, which raises important questions for clinical practice [33, 34].

Our study however poses several important limitations. HHV-6 positivity in bile did not significantly correlate with positivity in the liver biopsy. Even samples with very high virus counts in bile that where taken on subsequent days to the liver biopsy showed no virus amplification in the biopsy tissue. It could be possible, that the liver biopsy was non representative of the donor organ. The focal distribution of HHV-6 in infected liver tissue is not known. The design of the study was a retrospective case-control study wherein different cohorts were randomly selected from a tissue and fluid biobank. Concordant biopsy, serum and bile samples were not always available, and when they were available, they were not always taken on the same day.

The methods for DNA detection were not established for biliary fluids, and stability data on viral DNA in biliary fluid is lacking. Furthermore, viral loads for HHV-6 were low. Only 4 out of 25 patients had viral loads > 1000 copies/ml, the threshold for consideration as an active HHV-6 infection in serum samples [35]. While this finding could reflect a high percentage of latent infection and explain why viral DNA in those cases could not be detected in serum samples from the same recipients, this could also be due to DNA degradation in our bile samples. Our study warrants further exploration in a prospective experimental setting. If confirmed, treatment of occult HHV-6 and HHV-7 infection in cases of sustained and progressive biliary damage after LT might lead to a reduced risk of graft loss and need for re-LT. Further understanding of the role of non-CMV beta-herpesviruses in LT might also affect recommendations for the routine use of valganciclovir in the initial months after LT.

## Conclusion

The present study shows that CMV, EBV and HHV-6 and -7 are highly prevalent in biliary fluid. Overall beta-herpesvirus detection was more frequent in bile than in liver biopsy or serum. HHV-6 is associated with biliary complications after LT. Further studies are needed to assess the potential connection of HHV-6 detection in bile and biliary complications after LT and the general value of routine viral diagnostics in bile compared to serum and liver biopsy.

## Data Availability

The datasets used and/or analyzed during the current study are available from the corresponding author on reasonable request.

## Abbreviations

ACR: Acute cellular rejection
AS: Anastomotic biliary stricture
CMV: Cytomegalovirus
EBV: Epstein-Barr virus
eMELD: Exceptional model of end-stage liver disease
ERC: Endoscopic retrograde cholangiography
HCC: Hepatocellular carcinoma
HHV: Human herpesvirus
labMELD: Laboratory model of end-stage liver disease
LT: Liver transplantation
NAS: Non-anastomotic biliary stricture
PCR: Polymerase chain reaction
qPCR: Quantative polymerase chain reaction
SD: Standard deviation
Th1: T-Helper cell Type 1
VZV: Varicella-zoster virus
